# An approach to the etiology of metabolic syndrome

**Published:** 2013-03-30

**Authors:** Angélica M Muñoz Contreras, Gabriel Bedoya Berrío, Claudia M Velásquez R

**Affiliations:** a Research Group on Food and Human Nutrition. Universidad de Antioquia, E-mail: angelicamariamc@yahoo.com; b Faculty of Exact and Natural Sciences, Group of Molecular Genetics -GENMOL. Universidad de Antioquia, E-mail: gbedoya@quimbaya.udea.edu.co.; c School of Nutrition and Diet, Research Group on Food and Human Nutrition. Universidad de Antioquia, E-mail: claudia.velasquez@siu.udea.edu.co

**Keywords:** Metabolic syndrome, obesity, insulin resistance, genetics, ancestry

## Abstract

Increased prevalence of obesity in the world, especially accumulation of abnormal amounts of visceral fat predisposes to insulin resistance, which is the central role of metabolic syndrome (MS). Obesity can deregulate the intracellular signaling of insulin due to the production of inflammatory substances, chemoattractant proteins, adipokines and molecules that trigger hormonal mediator potentials for destabilization of signal transduction, leading to metabolic disorders such as hyperglycemia, hypertension, and dyslipidemia. The complexity of the MS and of the genetic mechanisms involved in its etiology derives from the combination of variants on genes involved and environmental factors that predispose it. The purpose of this paper is to review the effects of obesity in molecular and biochemical responses that trigger insulin resistance and its relation to some candidate genes and the ancestral component of the population.

## Abstract (es)

El aumento en la prevalencia de la obesidad en el mundo, especialmente el acumulo de grasa visceral, predispone a resistencia a la insulina, eje central del Síndrome metabólico (SM). La obesidad puede desregular la señalización intracelular de la insulina debido a la producción de sustancias inflamatorias, proteínas quimioatrayentes, adipoquinas y mediadores hormonales que activan moléculas potenciales para la desestabilización de la transducción de la señal, lo que genera alteraciones metabólicas como hiperglucemia, hipertensión y dislipidemia. La complejidad del SM y de los mecanismos genéticos involucrados en su etiología, se deriva de la combinación entre variantes génicas implicadas y factores del medioambiente que la predisponen. El propósito del presente artículo es revisar los efectos de la obesidad en las respuestas moleculares y bioquímicas que desencadenan resistencia a la insulina y su relación con algunos genes candidatos y el componente ancestral de la población.

## Introduction

Metabolic syndrome (MS) is a set of risk factors for Type-2 diabetes mellitus (T2DM) and cardiovascular disease (CVD), which includes abdominal obesity, hyperglycemia, dyslipidemia, and hypertension^1^. In 2009, a joint declaration was published among several organizations seeking to harmonize the diagnostic criteria of MS focusing on central obesity as a triggering element of insulin resistance (IR)^2^.

Obesity established by the World Health Organization (WHO) as the epidemic of the 21st century, is one of the most complex public health problems^3^; its prevalence has increased rapidly and it is a strong risk factor for numerous metabolic disorders that give rise to diseases like diabetes, fatty liver, cancer, and cardiovascular disease, among others^4^.

Insulin resistance associated to obesity is considered the fundamental cause of MS. The development of diseases comprising it is the result of genetic susceptibility due to the combination of variants in genes implied in the homeostasis of energetic metabolism and the interaction of said variants with appropriate environmental factors for its expression. Although numerous studies have been conducted on the genetic etiology of MS, only a few genes have been identified with moderate and replicable effects in populations from different ancestries, which indicate that risk variants have different frequencies in continental populations. With these approaches, it is clear that addressing the etiology of MS must be done via comprehensive analyses of the genetic and environmental aspects that predispose it.

## Central obesity, triggering condition of insulin resistance

The lack of balance between nutrient intake and energy expenditure, associated with genetic susceptibility explains the onset of the phenotype of obesity. To date, 32 loci have been identified via genomic scans with association to risk of obesity^5^. The gene "fat mass and obesity-associated" (FTO), the first identified through this methodology, is currently the one that has shown the strongest association and high consistency. Adults who weighed nearly three Kg more compared to those who did not present the variant reported^6^. Identification of the variants found in the genomic scans has been verified by other studies, both in adults as in children^7^ ^,^ ^8^, revealing cumulative effects for the body-mass index (BMI)^7^; some of these variants have been associated with anthropometric variables like the waist-hip ratio, but after adjusting for BMI these lose association^9^. This suggests that said loci are important in determining body fat gain in general, but not in its distribution. These loci are probably implied in many pathways because the variants are expressed in numerous tissues; however, most are primarily found in the hypothalamus, which is strongly implied in weight regulation and nutrient intake^10^. Bauer *et al*., found association between the dietary intakes of specific macronutrients: proteins, fats, and carbohydrates and some of the variants found^9^.

In obese subjects, especially those with central-type obesity due to accumulation of visceral fat, the urgency to metabolize the overload of nutrients from excessive intake and low energy expenditure associated to poor physical activity subjects the cells to metabolic stress that initiates and perpetuates oxidative and inflammatory cascades, leading to damage in insulin signaling and resistance of tissues to hormone action. Excess of visceral fat, more than subcutaneous fat, is associated to IR because of the metabolic peculiarities of this type of fat as the greatest tendency to lipolysis, increased activity of the glucocorticoid receptor, greater secretion of inflammatory cytokines, and reduced secretion of insulin sensitizing adipokynes. Also, the physical location of visceral fat permits liberating free fatty acids (FFA) and other metabolites of the adipose tissue directly onto the portal circulation and from there its direct ingress to nearby organs like the liver and pancreas, which are exposed to lipotoxicity^11^. Recently, two loci were identified, which influence on central adiposity: near the transcription factor AP-2 beta (TFAP2β) and methionine sulfoxide reductase A (MSRA) and one in the fat distribution only in women: lysophospholipase-like-1 (LYPLAL1)^12^. The loci near TFAP2B were indicated as the most obvious candidate gene; its over-expression down-regulates expression of the insulin-sensitizing hormone adiponectin by direct transcriptional repression. Conversely, no direct relationship was found in the connection between the MSRA locus and adiposity; however, its closeness to the tankyrase gene (TNKS), which encodes a telomeric-repeat binding factor (TRF1) -interacting ankyrin-related ADP-ribose polymerase. Tankyrase, a peripheral membrane protein that interacts with aminopeptidases and responds to insulin, was related to disposition of glucose within the fatty tissue. The signal near to LYPLAL1 corresponds to the lysophospholipase protein acting as a triglyceride lipase and correlating positively with the subcutaneous adipose tissue of obese subjects.

The details of obesity as a causal factor of IR are complex; awareness of the molecular aspects of insulin signaling will facilitate its understanding.

## Molecular aspects of insulin signaling

After food intake, glucose levels in plasma increase. This rise is detected by ß-cells in the pancreas, which secrete insulin, principal regulator of metabolism; this hormone reduces glucose levels in plasma^13^; stimulates synthesis of proteins, fatty acids and of molecules implied in cell growth and repair; as a signaling molecule, it sends information to the brain and to the central nervous system on the peripheral energetic availability^14^.

**Figure 1:**
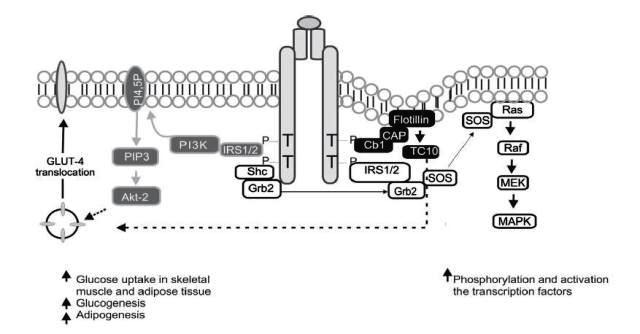
Molecular pathways of insulin signaling. Grey background: phosphatydil-inositol-3 kinase (PI3K) signaling pathway; black background: CAP/Cbl/TC10 pathway; White background: MAP-Kinase dependent

The metabolic effects of insulin in peripheral tissues, such as glucose transport are mediated by a cascade of intracellular signals. Insulin starts its action by binding its receptor with endogenous tyrosine kinase activity, formed by a tetrameric transmembrane protein, consisting of two ∞- and two ß-subunits. After binding of insulin, a rapid autophosphorylation of the receptor occurs in diverse residues of tyrosine of the ß sub-units; acquiring tyrosine kinase activity that can modify other substrates. Among the intracellular substrates used by these receptors, six belong to the insulin receptor substrate family (IRS1-6)^15^, which act as docking molecules for proteins containing SH2 domains, while permitting downstream signal transmission; IRS1 and IRS2 are ubiquitously expressed and are the main mediators of insulin-dependent mitogenic activity and of the regulation of glucose metabolism in most cell types^16^; other substrates are the isoforms of adapting proteins Cbl, Shc, Gab1/2, and members of the APS family (adapting proteins with PH and SH2 domains). The effectors that have been characterized to bind IRS proteins include phosphatidyl-inositol-3-kinases (PI3K), Growth factor receptor-bound protein 2 (Grb-2), SH2 domain-containing tyrosine phosphatase (SHP-2), Fyn, cellular CT10 regulator of kinase (c-Crk), among others, all of which intervene as mediators in metabolic functions and in insulin growth promoter functions^17^. The proteins, intracellular substrates, which bind the phosphorylated receptor may activate the signaling of the PI3K pathway, CAP/Cbl/TC10 pathway, and the mitogen-activated protein kinase (MAPK)^18^, as shown in Figure 1.

The effects of insulin in glucose metabolism are mediated, in part, by IRS-1, which contains from 20 to 22 phosphorylation sites in tyrosine and over 30 phosphorylation sites in serine or threonine^19^. The activated IRS interacts with SH2 regulatory domains of PI3K that yield the activation of this enzyme; the PI3K catalytic sub-unit is located near the plasma membrane where it has access to the phosphatydil-inositol 4,5-biphosphate substrate (PI4,5-P2), which induces the production of phosphatydil-inositol-3,4,5 tri-phosphate (PIP3); this last, in turn, activates kinase dependence of PI3K (PKB), which at the same time activates the serine/threonines protein kinase Akt-2, which acts as a central mediator of the effects of insulin on the intermediate metabolism, including glucose uptake and synthesis of glycogen and fatty acids^20^.

Parallel to this, an alternative pathway is activated required for glucose uptake by extra hepatic tissue, especially in adipocytes, through phosphorylation of the Cbl protein, associated with the CAP adapter protein; this complex is translocated with the intervention of the flotillin protein that interacts with the SoHo domain of CAP, once translocated into a lipid domain denominated rafts in the plasma membrane, activates the G protein TC10 that provides a second signal for the translocation of Glucose transporter type 4 (GLUT4)^18^. The third pathway, the MAP-kinase dependent, starts when Grb2 attaches Shc or IRS to the SH2 domain of the Grb2 protein, which is attached to Son of Sevenless (SOS), a small nucleotide exchange protein that catalyzes guanosine di-phosphate (GDP) exchange by guanosine tri-phosphate (GTP) in the protein found in the internal surface of the plasma membrane called Ras - such also favors Raf phosphorylation in serine/threonine sites. The Raf molecule stimulates MEK, leading to the activation of MAP kinase and cellular proliferation and differentiation via regulation of genetic transcription^18^ ^,^ ^20^

## Possible molecular mechanisms involved in the alteration of insulin signaling by obesity

Diverse mechanisms are implied in the inhibition of the transduction of the insulin signal. A possible mechanism is IRS phosphorylation in the serine and threonine amino acids and not in tyrosine; this phosphorylation may induce the dissociation of IRSs from IR, hinder or diminish IR phosphorylation in tyrosine, or induce the degradation of IRS by the proteosome^21^ ^-^ ^23^. In addition to the previous mechanisms, studies have shown that IRS1 and IRS2 interact with cytosolic transport proteins 14-3-3 through phosphoserine residues within the PTB domain and this binding may physically prevent the interaction with the insulin receptor^24^. Some potential candidates for IRS desensitizing are the mammalian target of rapamycin/Ribosomal protein S6 kinase beta-1 (mTOR/S6K1), c-Jun-N-terminal Kinase (JNK), multiple members of the protein kinase C (PKC) family, salt-inducible kinase 2 (SIK-2), which enhance the phosphorylation of serine residues in the carboxyl terminal^25^ ^,^ ^26^. Another kinase, part of the desensitizing of the insulin signal, is inhibitor kinase kappa ß (IKKß) that activates the nuclear transcription factor -Kß (NF-Kß)^27^. Also, the family of suppressors of cytokine signaling (SOCS), through direct or indirect interactions plays an important role in the negative regulation of the IRS activity, altering their structure and interaction, both for the insulin receptor and the PI3K-type effector proteins^28^.

**Figure 2:**
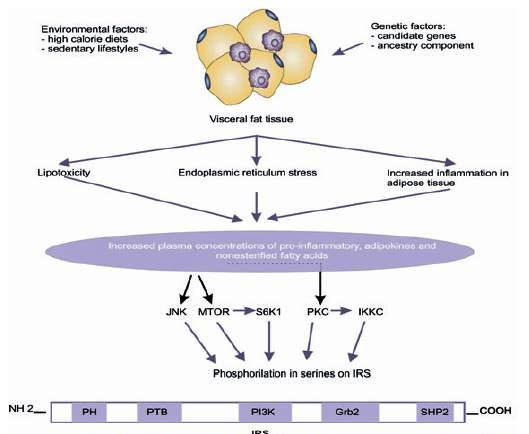
Negative IRS regulation. Environmental and genetic factors intervene in the alterations of visceral adipose tissue increasing secreting substances that lead to phosphorylation in IRS1 serine by activating intracellular signals.

Additionally, exogenous factors like obesity may activate serine/threonine kinases that phosphorilate IRS1 and inhibit its function. In obese individuals, the adipocyte is altered by fat overload and generates three harmful effects: 1) an inflammatory response that starts with hypertrophy and hyperplasia of the adipose tissue is propagated by infiltration of activated macrophages and is exacerbated by the production of adhesion molecules; 2) stress of the adipocyte endoplasmic reticulum by overload in its functional capacity, and 3) increase of circulating levels of non-esterified FFA and glycerol, leading to an increase in the disposition of those lipids within peripheral organs not suited for fat storage like the liver and pancreas, where the non-esterified FFA become the most probable cause of the damage of the Kß-cell function, given that chronic exposure is associated to damage in the secretion and decrease in biosynthesis of insulin stimulated by glucose^29^, which is known as lipotoxicity (Fig. 2). These three elements converge into a great production of inflammatory and chemokine substances such as the tumor necrosis factor-alpha (TNF-∞), interleukin 6 (IL-6), IL-1Kß, and monocyte chemoattractant proteins (MCP-1); there is also greater production of other adipokynes directly implied in thrombosis and hypertension like the plasminogen-1 (PAI-1) inhibitor/activator, angiotensinogen, endothelin; lipid mediators like prostaglandins, lipoxins and resolvins, among others that participate in the activation of inflammatory cells, growth, development, and dysfunction of the adipocyte and of hormonal mediators that alter adequate insulin signaling, inducing changes in glucose metabolism, coagulation, fibrinolysis and inflammation, which modify the cardiovascular structure and function and lead to endothelial dysfunction and atherosclerosis^30^ ^,^ ^31^.

The mechanisms previously exposed, through which obesity deregulates the insulin signal, act via activation of Ser/Thr kinases, possibly the same that are involved in the physiological control of insulin signaling. Thus, for instance, FFA increase leads to the increment of certain intracellular metabolites, like diacylglycerol, Acil-CoA, and ceramides, which can activate Ser/Tre kinases (protein kinase CØ) that phosphorilate IRS 1 and 2 in Ser/Tre sites - reducing the receptor's ability to initiate the PI3k pathway and, thus, the decrease of glucose transport^32^; TNF∞ can induce the activation of ceramide production, which can activate PKC and with such IKKKß; the activity of the kinase c-Jun amino terminal (JNK1) has been related to FFA, the TNF-∞ that phosphorilates IRS-1 in the Ser307, decoupling the IR^33^; in addition to other metabolic stress factors, like free radicals, hyperglycemia and hyperlipidemia^34^.

## Insulin resistance, central focus of metabolic deregulation

Insulin resistance leads to greater hepatic production of glucose as if the organism were fasting; however, levels of blood glucose are increased. The organism compensates by increasing insulin secretion, which generates a state of hyperinsulinemia. Hyperglycemia occurs only in those individuals able to secrete sufficient compensatory insulin^35^. Obesity and IR are associated to T2DM when the 1Kß-cells are incapable of completely remediating the reduced sensitivity to insulin. Besides hyperglycemia, other metabolic alterations like dyslipidemia occur, which includes homeostatic alteration of fatty acids, triglycerides, and lipoproteins; damage in the central regulation of intake and satiety due to deterioration in the integration of signals related to the energetic situation of the organism and hypertension by the activation of the sympathetic nervous system and the renin-angiotensin system^36^.

Vascular diseases and diabetes consequential of MS have been additionally associated to genetic susceptibility. In the case of studies of genomic scans for T2DM, over 20 susceptible loci have been associated^37^. When the ß-cell functions adequately, the adaptive response to insulin resistance implies changes in its mass and function, and it is so efficient that the normal tolerance to glucose is maintained. This essential function is carried out through a feedback system in which glycemia regulates the increase of the ß-cell function among them. For cellular expansion to take place, an increase is required of the peptide signals similar to glucagon 1 (GLP-1) that promotes proliferation, neogenesis and prevents apoptosis of ß-cells^38^. The glucotoxic effect of prolonged hyperglycemia is one of the stimuli that keep these signals from carrying out effective cellular regeneration, producing increased cellular death by apoptosis.

The lipotoxic effect may also act in synergy with glucose to produce greater damage, commonly referred to as glucolipotoxicity^39^. A gene codifying for the transcription factor 7 and 2 (TCF7L2) has been related as a risk factor for T2DM. The product of the TCF7L2 gene is the transcription factor 4 of T cells (TCF-4), this mobilization of transcription factors plays an important role in the pathways that involve Wnt, a protein that triggers a cascade of intracellular signals in charge of activating the signals from the GLP-1 peptide, responsible for regulating the mechanisms of development and growth of the ß cells in the pancreas^40^. The presence of the variant in the Single Nucleotide Polymorphism (SNP) rs7903146 in the TCF7L2 gene confers a risk T allele, which is associated with diminished insulin secretion^41^. Another of the representative genes found is solute carrier family 30 (zinc transporter) member 8 (SLC30A8), which codifies for the Zinc 8 transporter. It is found in the plasma membrane of the β-cell and permits the entry of the ion to accumulate along with insulin in the secretory granules. A variation in the gene produces alteration in the accumulation of zinc and causes a change in insulin stability^42^.

Additionally, hypertension and dyslipidemia are frequent co-morbidities in patients with insulin resistance. Insulin fulfills an important function in the process of regulating blood pressure, acts as a vasodilator by the liberation of nitric oxide from the vascular endothelium, and facilitates tubular re-absorption of sodium through the endothelial action of the Angiotensin Converting Enzyme (ACE) system^36^. In healthy subjects, the vasodilatory and pressor effects are compensated; in physiopathological states like obesity, the equilibrium may be broken by increasing the sympathetic activation in response to hyperinsulinemia and elevating the activity of the rennin-angiotensin system, which may increase vasoconstriction, leading to increased blood pressure. Evidence of genetic influences in the development of hypertension comes from different sources; a large number of candidate genes and independent signals reaching genome-wide significance have been tested for association with hypertension^43^. The mechanisms by which insulin resistance induces atherogenic dyslipidemia, characterized by elevated circulating levels of very-low-density lipoprotein (VLDL) and small and dense low-density lipoprotein (LDL), with sub-normal levels of high-density lipoprotein (HDL) are related to the hepatic accumulation of fatty acids, which promote triglyceride synthesis and production of VLDL^44^.

The lipoprotein lipase (LPL) of the adipose tissue promotes the efficient uptake and storage of FFA from lipoproteins, the production and activity of this enzyme is stimulated by insulin; insulin resistance diminishes the LPL expression in the adipocyte and with this the storage and uptake of fatty acids remaining free in plasma^45^. Also, the cholesterol ester transporting protein (CETP) mediates the transference of esters of HDL cholesterol for esterified triglycerides in VLDL and LDL, besides contributing directly in the formation of small and dense LDL particles. During a parametric linkage analysis in 59 families from China with early onset hypertension, a link was detected to two chromosome regions that contained the LPL and dipeptidyl carboxipeptidase 1 (DCP1) genes; in the analysis, the greatest signal was for the region with the LPL gene^46^, which evidenced the association between dyslipidemia and hypertension, then this relationship was confirmed with a recent study associating 95 loci with high concentrations of lipids in plasma and among which the variants of the LPL and CEPT genes were identified^47^.

Based on these findings, the study of the MS etiology must involve variants in the genes related to the pathways of insulin signaling, as well as other stimulating and inhibiting molecules and external factors to these pathways, whose participation is given in the cascade of cellular events. 

Although there are reports of nearly 40 Genome-Wide Association Studies (GWAS) in relation to MS traits, none of the studies conducted have included individuals of mestizo origin. Reports available regarding association of regions or genetic variants in Amerindian populations is quite limited although on the rise^48^.

In 2007, a variant in the ATP binding cassette transporter 1 (ABCA1) gene was found in the Mexican population, implied in the formation of HDL, which was associated with obesity and T2DM in this population^49^. A recent study confirmed an in vitro functional effect of the variant studied (R230C, rs9282541)^50^; likewise, the LPL gene was also associated to dyslipidemia, with RI^51^. The Calpain 10 genes (CAPN10) have been originally identified through linkage studies associated with T2DM and obesity; variants were associated to subclinical atherosclerosis and factors determining a pre-diabetic phenotype in a non-diabetic Mexican-American population. In Chilean individuals, the polymorphism pro72Arg of the TP53 gene was associated with coronary disease in a study of cases and controls; however, this association is suggested to be confirmed with a population study^52^. Moreover, in a population from the Colombian northeast, variants of uncoupling proteins 1, 2, and 3 and their haplotypes conferred risk for T2DM^53^. In addition, studies indicate that the effect of polymorphisms on the risk of T2DM and CVD related to insulin resistance depends on the clinical characteristics of the individuals, including body weight and age of onset of the disease^54^.

## Role of the ancestry component in etiology of metabolic syndrome

In studies conducted to determine the hereditary burden of MS and its associated traits, it was reported that MS is most frequent in Hispanics compared to Afro-Americans or non-Hispanic Whites in the USA^55^. Also, the prevalence of the different components of MS reveals ethnic differences, HTA is most frequent among African Americans and West Africans than among Europeans^56^; dyslipidemia is most frequent among Europeans, the Black population has favorable lipid profiles in spite of having greater risk of obesity and hypertension^57^, and T2DM is more prevalent among Amerindians^58^. Recently, in Puerto Rico, with an admixed population, the African ancestry correlated positively with the prevalence for hypertension^59^. Furthermore, fasting glucose was greater among Hispanics in comparison to Afro-Americans and non-Hispanic Whites in the USA^60^. As can be noted, differences exist among the ancestral continental populations of Latin American populations like the Colombian population (Amerindian, European, and African). The combinations of the gene variants, the environmental factors, and the demographic history are different among the diverse ancestral populations; hence, when seeking to study the etiology of MS in admixed populations it is important to adjust by ethnicity, given that it has been found that the association presented may be due to ethnicity and not to the status of the disease because of the difference in ancestral composition in case groups and controls.

In conclusion, obesity and the metabolic alterations generated by MS are explained by the interaction of multiple genetic and environmental predisposing factors. Particularly, the genetic etiology of these diseases shows concurrence in an individual from the combination of multiple variants and ancestral determinants predisposing to the phenotype. Consequently, research inquiring on the etiology of MS, as a disease of complex genetics must comprehensively consider genetic aspects like the variants in candidate genes, the ancestral genetic composition; environmental aspects like food intake and the degree of physical activity and individual characteristics like gender, age, body composition, and family antecedents of disease.
